# Transanal sphincter-sparing local excision for primary anorectal malignant melanoma: a case report

**DOI:** 10.3389/fonc.2026.1730918

**Published:** 2026-03-19

**Authors:** Zhiting Wang, Jianwen Hu, Yingfeng Xu, Shiwei Chen

**Affiliations:** 1 Department of Anorectal Surgery, The Seventh Clinical College of Guangzhou University of Chinese Medicine, Shenzhen, China; 2 Department of Traditional Chinese Medicine, Affiliated Traditional Chinese Medicine (TCM) Hospital of Guangzhou Medical University, Guangzhou, China

**Keywords:** anorectal, case report, malignant, malignant melanoma, melanoma, primary, wide local excision

## Abstract

**Background:**

Anorectal malignant melanoma (ARMM) is a rare and highly aggressive malignancy with a poor prognosis. Surgical resection remains the primary treatment strategy, with local excision (LE) and extended resection (ER) being the most commonly performed approaches. However, the optimal surgical management remains controversial, particularly regarding oncologic control and sphincter preservation.

**Case presentation:**

We report a 61-year-old man who presented with painless hematochezia for one month. Imaging revealed a 4 × 4 cm mass near the dentate line, and colonoscopic biopsy confirmed malignant melanoma. After multidisciplinary evaluation, transanal sphincter-preserving local excision was performed under combined spinal-epidural anesthesia. The tumor was resected with preservation of anal function, and the postoperative course was uneventful without fecal incontinence. However, the patient died 14 months after surgery.

**Conclusion:**

This case illustrates that transanal sphincter-sparing local excision may be a feasible surgical option for carefully selected patients with localized primary anorectal malignant melanoma without sphincter involvement or distant metastasis. Nevertheless, longer follow-up and further studies are required to evaluate long-term oncologic outcomes.

## 1 Introduction

Anorectal malignant melanoma (ARMM) is an extremely rare and aggressive malignancy, including both primary and secondary forms, accounting for approximately 1% of all anorectal cancers (1, 2). It typically arises from melanocytes located in the mucosal epithelium near the dentate line. The exact pathogenesis remains unclear but may involve alterations in the mucosal microenvironment together with genetic mutations (3, 4). (Cite the relevant references). Surgical resection remains the cornerstone of treatment, with the most common approaches including local excision (LE) and extended resection (ER), with ER encompassing procedures such as abdominoperineal resection (APR) (1, 5). LE includes wide local excision (WLE), which removes the tumor with a safe margin while preserving sphincter function, whereas ER involves more extensive resections that may compromise anal function. Due to the high risk of distant metastasis and poor prognosis, even radical surgery rarely leads to long-term survival benefits (6). In recent years, sphincter-preserving local excision techniques have gained attention for their ability to balance oncologic safety with preservation of anal function and quality of life (7). (Cite the relevant references). Here, we report a case of primary anorectal malignant melanoma (PARM) successfully treated with WLE, and, based on the existing literature, discuss its feasibility, clinical value, and the treatment strategies for this disease.

## 2 Case report

### 2.1 Clinical presentation and medical history

**Figure 1:**
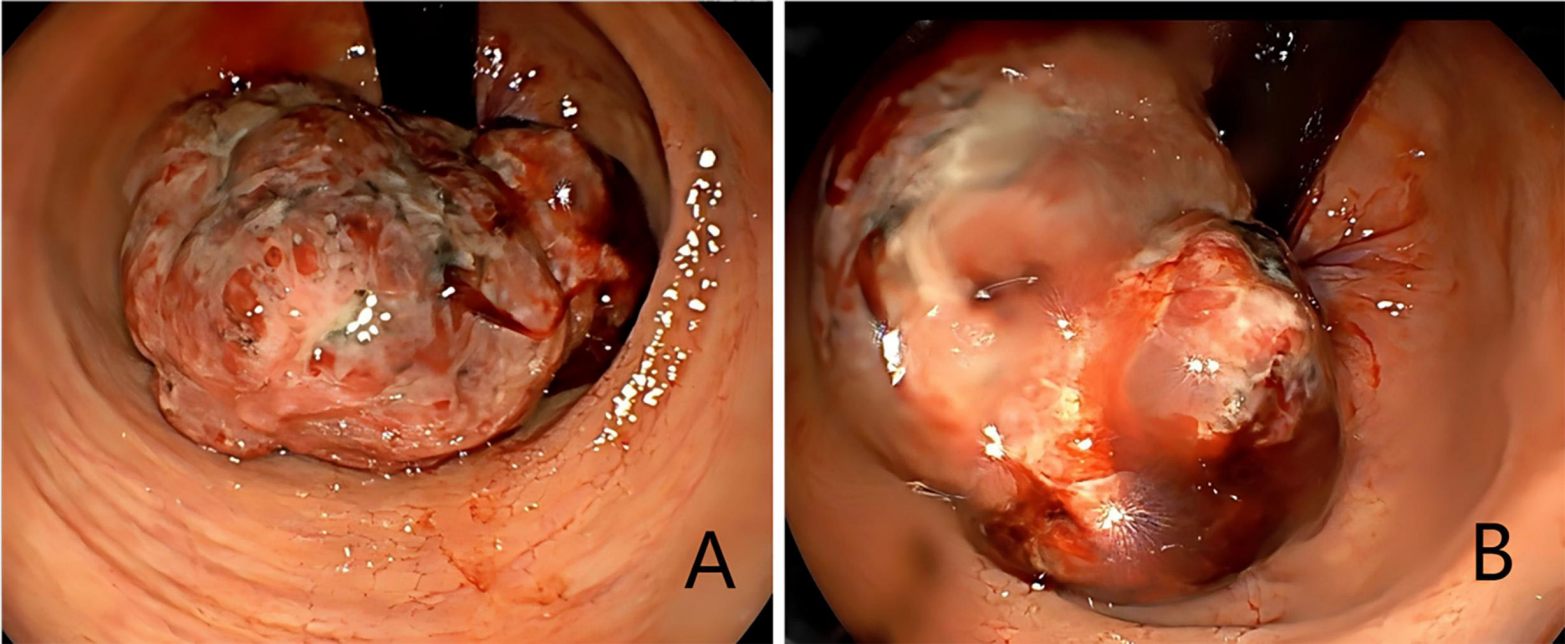
**(A, B)** Colonoscopic view of the mass, measuring approximately 4 × 4 cm and located about 3 cm from the anal verge, showing surface erosion.

**Figure 2:**
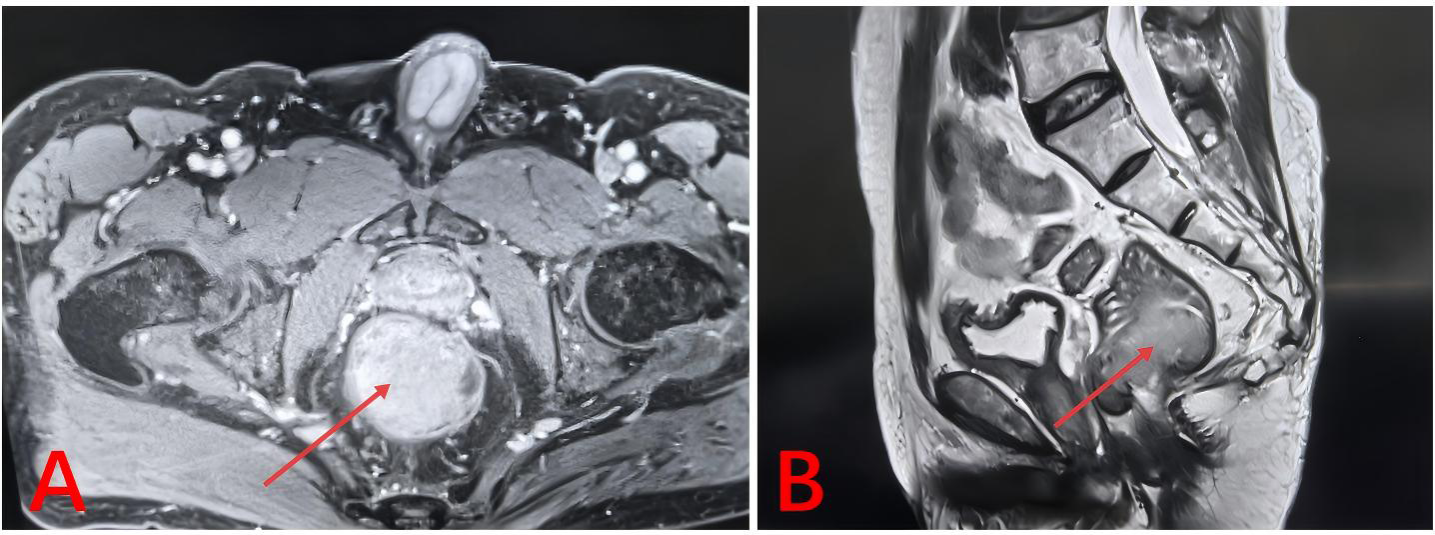
3T rectal and pelvic MRI with and without contrast enhancement. The red arrow indicates the location of the tumor, measuring approximately 45 × 34 × 48 mm. **(A)** Axial view; **(B)** Sagittal view.

**Figure 3:**
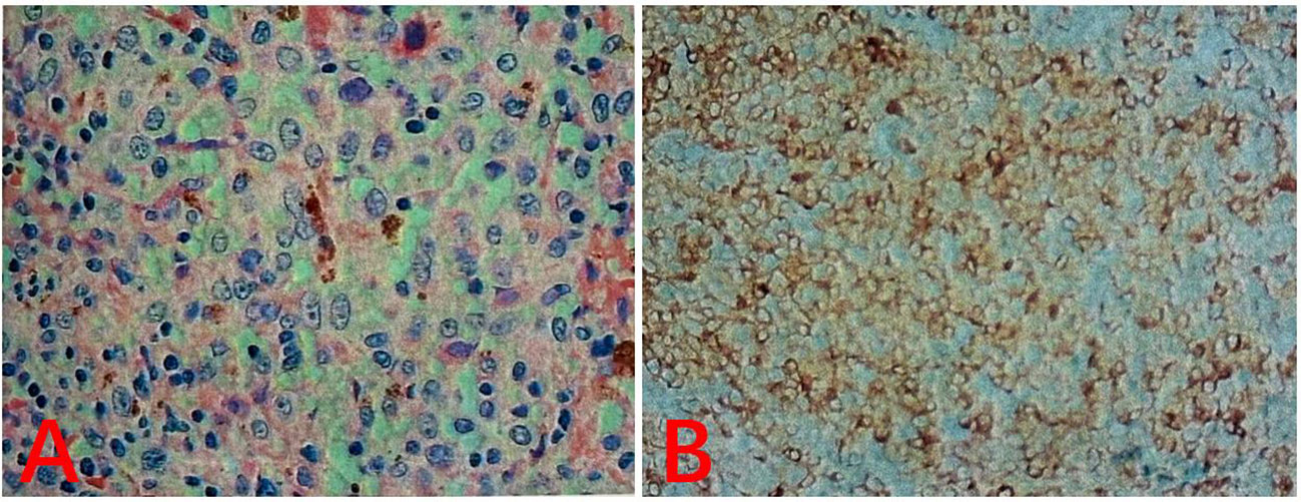
**(A)** Hematoxylin and eosin (H&E) staining: The tumor was composed of medium-sized round, epithelioid, or spindle-shaped cells with abundant eosinophilic cytoplasm. A small number of cells contained intracytoplasmic pigment granules. The nuclei were large and round, and prominent nucleoli were observed in some cells. **(B)** Immunohistochemistry: S-100 (−), HMB45 (partial +), Melan-A (+), CK (−), SOX-10 (+), CK20 (−), CDX2 (−), and Vimentin (+) .

A 61-year-old male presented with a one-month history of painless rectal bleeding. The blood was bright red and drip-like, accompanied by anal discomfort and a sensation of prolapse. The patient reported mild dizziness and fatigue but no weight loss. His body mass index (BMI) was 24.8. He had no history of hypertension, diabetes, or familial malignancies, but reported a history of smoking and alcohol consumption. (Add body mass index, smoking history, and alcohol consumption history). Digital rectal examination revealed an irregular, firm mass approximately 4 cm from the anal verge, located between the 5 and 9 o’clock positions, without bleeding on glove withdrawal. Colonoscopy revealed an irregular mass located approximately 4 cm from the anal verge, extending to the anal canal and dentate line. The surface was uneven and friable, with contact bleeding, and the lesion protruded into the rectal lumen (**Figure 1**). Contrast-enhanced pelvic MRI demonstrated irregular rectal wall thickening and a soft-tissue mass measuring 45 × 34 × 48 mm below the peritoneal reflection, involving the rectal lumen over a length of 48 mm and located 42 from the anal verge (**Figure 2**). No pelvic lymphadenopathy or adjacent organ invasion was observed. CT scans of the chest and abdomen showed no distant metastases. Laboratory tests revealed mild anemia, with normal tumor markers, coagulation, and biochemical profiles. Histopathology from colonoscopic biopsy confirmed malignant melanoma. Immunohistochemistry results were as follows: S-100 (-), HMB45 (partial +), Melan-A (+), CK (-), SOX-10 (+), CK20 (-), CDX2 (-), Vimentin (+) (**Figure 3**). The unusual S-100 negativity is noted and discussed below.

### 2.2 Surgical procedure

**Figure 4:**
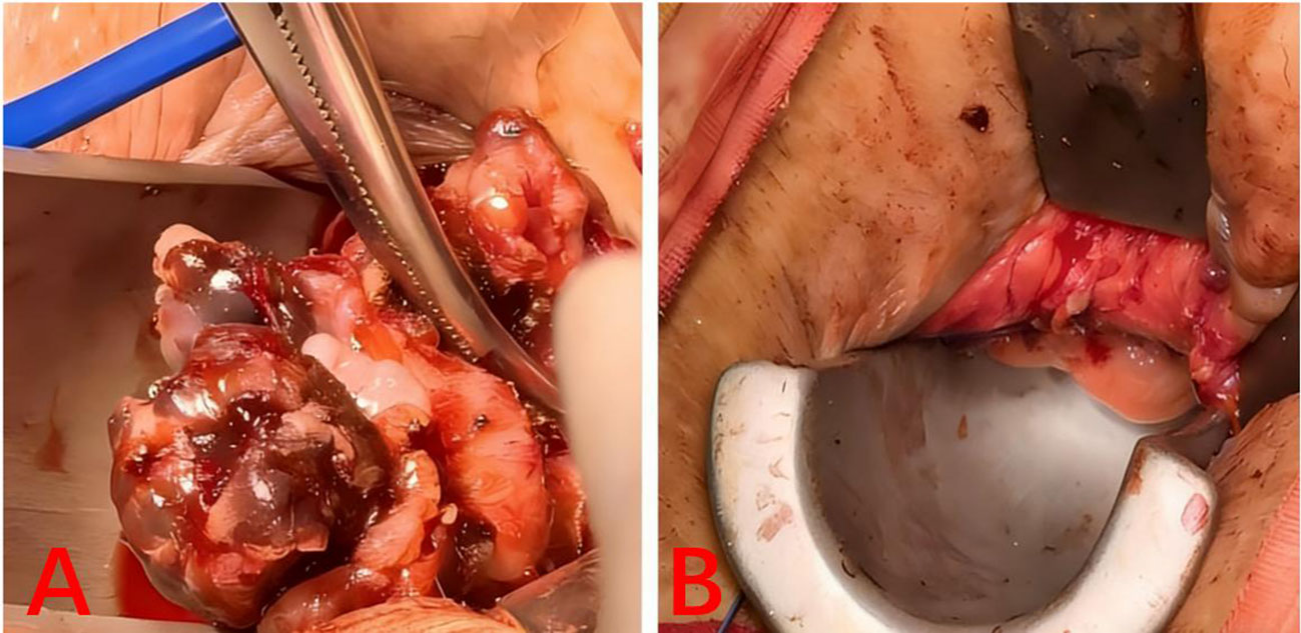
Intraoperative gross specimen of the tumor; **(A)** Intraoperative view of the anal tumor; **(B)** Postoperative appearance of the anus.

Although anorectal melanoma is highly aggressive, this patient’s lesion was localized without evidence of metastasis, and he expressed a strong desire to preserve anal function. Following multidisciplinary team (MDT) discussion, APR and partial rectal resection were ruled out. To ensure complete tumor removal while maintaining postoperative quality of life, wide local excision (WLE) (Clarify the surgical approach) was selected (**Figure 4**).

Under combined spinal-epidural anesthesia, the patient was placed in the lithotomy position. An anoscope (Modify “transanal endoscope” to “anoscope.”)was used to visualize the tumor. A circumferential mucosal incision was performed approximately 0.5 cm from the tumor margin using an ultrasonic scalpel, beginning at the 6 o’clock position and extending laterally. Careful dissection was carried out along the mucosal and partial muscular layers to facilitate complete tumor removal. Intraoperatively, the tumor appeared exophytic, friable, and partially necrotic, occupying approximately 50% of the rectal lumen. An en bloc resection was initially attempted; however, due to the tumor size and fragile consistency, complete single-piece excision was not feasible. The lesion was therefore removed in a piecemeal fashion. The surgical defect was closed with absorbable sutures at the 3, 6, and 9 o’clock positions. All specimens were submitted for histopathological evaluation. Estimated blood loss was 10 mL, and there was no injury to the sphincter or nerves. The postoperative course was uneventful. The patient had normal bowel movements by postoperative day 3 without fecal incontinence or anal pain. Pathologic examination revealed gray-brown friable tumor fragments measuring 6 × 6 × 2 cm, with nests and sheets of atypical tumor cells, nuclear pleomorphism, and areas of melanin pigmentation and multinucleated giant cells. Because the specimen was fragmented, margin assessment was inconclusive, and close postoperative surveillance was recommended.

### 2.3 Postoperative follow-up

One month after surgery, the patient underwent further immunohistochemical and molecular analyses (including FISH and HER-2/neu testing) at a local hospital, confirming the diagnosis of anorectal melanoma, consistent with the preoperative colonoscopic pathology, and ruling out melanoma-like sarcomas such as clear cell sarcoma (CCS). Under the recommendation of the oncologist, the patient received adjuvant chemotherapy with paclitaxel, carboplatin, and bevacizumab. The regimen was scheduled to be administered every three weeks. However, the patient completed only two cycles and subsequently discontinued treatment, without proceeding with the remaining planned therapy. At the 14-month postoperative follow-up, we were informed that the patient had died. According to his family, the cause of death was attributed to widespread systemic metastases. Owing to the lack of complete terminal clinical records, the detailed course of disease progression and the exact circumstances of death could not be further verified. (to incorporate the patient’s perspective).

## 3 Discussion

ARMM is a rare but highly aggressive malignancy, accounting for approximately 1% of all melanomas, and it represents one of the more common sites of melanoma arising in the gastrointestinal tract (8). Due to the lack of specific early symptoms, such as painless rectal bleeding, changes in bowel habits, and anal discomfort or a sensation of rectal fullness, the disease is often misdiagnosed as hemorrhoids or anal fissure, leading to delayed diagnosis and poor prognosis (2, 9). Some studies have reported that the 5-year survival rate is only approximately 10%–20% (10), and the median survival time in most patients is typically less than 2 years (11).

### 3.1 Surgical treatment: radical resection vs. function preservation

Surgery remains the primary treatment modality for localized ARMM; however, due to its high rates of local recurrence and distant metastasis, the optimal surgical approach remains controversial (12). Traditionally, APR has been regarded as the standard radical procedure. Nevertheless, recent studies have demonstrated that for localized lesions without distant metastasis, WLE can achieve survival outcomes comparable to those of APR, while significantly reducing postoperative sphincter dysfunction and improving quality of life (13, 14). Therefore, WLE may represent a reasonable balance between oncologic control and functional preservation.

However, in ARMM characterized by high invasiveness and metastatic potential, WLE requires precise preoperative staging (including MRI, PET/CT, and endoscopic evaluation) as well as meticulous intraoperative technique. This procedure is only appropriate when the sphincter is not involved and no lymph node or distant metastasis is present. Achieving negative surgical margins (R0 resection) is critical for long-term outcomes. In cases where the specimen is fragmented or the margins cannot be clearly assessed, intraoperative frozen-section analysis and regular postoperative imaging follow-up are essential for ongoing management.

### 3.2 The importance of preoperative assessment and postoperative pathology

Accurate preoperative evaluation is crucial in the management of ARMM. Endoscopic examination can determine the tumor’s location, size, depth of invasion, and its relationship with the dentate line. Magnetic resonance imaging (MRI) is the primary modality for assessing the extent of local tumor invasion and its relationship with surrounding structures, and it is particularly useful for evaluating sphincter and adjacent tissue involvement. Positron emission tomography/computed tomography (PET/CT) may be used to assess distant metastasis. Comprehensive preoperative evaluation facilitates appropriate surgical planning. Molecular pathology plays an important role in precision oncology, helping to identify potential therapeutic targets and exclude histologic mimics. S-100 protein is one of the most commonly used and highly sensitive immunohistochemical markers for the diagnosis of melanoma. In most melanomas, including both cutaneous and mucosal types, S-100 is typically strongly positive; therefore, it is considered a fundamental screening marker in pathological diagnosis. In patients with ARMM, only approximately 10%–20% of melanomas are S-100 negative (15). Therefore, S-100 negativity does not exclude the diagnosis of melanoma but indicates the need to expand the panel of immunohistochemical markers. Although S-100 is highly sensitive, it is not specific for melanoma (16). In contrast, markers such as HMB-45, MART-1/Melan-A, tyrosinase, and MITF are less sensitive than S-100 but generally demonstrate higher specificity (17, 18).

### 3.3 Current status and selection of adjuvant therapy

Given the extremely high risk associated with ARMM, surgery alone is often insufficient to significantly improve long-term survival. Currently, there is no consensus regarding the optimal choice of adjuvant therapy. Radiotherapy may be considered for local control in high-risk patients or in cases with close/positive surgical margins. Some studies suggest that radiotherapy and chemotherapy may reduce the risk of local recurrence; however, existing evidence has not demonstrated a significant improvement in overall survival (19). In recent years, targeted therapy for melanoma has made substantial progress, particularly in cutaneous melanoma. Targeted treatments may benefit selected patients harboring BRAF or KIT mutations. However, mucosal melanoma (including anorectal melanoma) has a molecular profile that differs from that of cutaneous melanoma. Therefore, there is a lack of universally applicable, evidence-based standardized targeted treatment strategies for this subtype (20, 21). Similarly, immunotherapy has demonstrated potential benefits in some patients with mucosal melanoma; however, its overall efficacy is less well established compared with that in cutaneous melanoma (22, 23). To further characterize the molecular profile and explore potential targeted therapeutic options, the patient underwent HER-2/neu genetic testing, which revealed no evidence of HER-2 gene amplification or overexpression. In the absence of identifiable actionable molecular alterations, and considering the tumor’s high aggressiveness, the inability to definitively assess negative surgical margins intraoperatively, and the elevated risk of postoperative recurrence, the oncologist recommended adjuvant chemotherapy with paclitaxel and carboplatin combined with bevacizumab. This regimen was selected based on its cytotoxic effects and anti-angiogenic mechanism, aiming to enhance systemic disease control and reduce the risk of recurrence as part of a comprehensive treatment strategy.

It should be emphasized that the patient died 14 months after surgery. Owing to the lack of complete terminal clinical documentation, the exact mechanism of death could not be definitively determined. According to the family, the patient died of widespread systemic metastases. Given the highly aggressive nature of ARMM and its propensity for early dissemination, disease progression was likely a major contributor to the unfavorable outcome. This case suggests that in the presence of high-risk factors—such as uncertain surgical margin status and incomplete adjuvant therapy—patients may face an increased risk of recurrence and metastasis. These findings further underscore the importance of standardized postoperative comprehensive treatment and long-term follow-up management.

## 4 Conclusion

Anorectal malignant melanoma is a rare and highly aggressive malignancy characterized by difficulties in early diagnosis and poor prognosis. For carefully selected patients with localized disease, wide local excision, provided that negative margins are achieved, may offer a reasonable balance between oncologic control and functional preservation. This case highlights the importance of clearly defined surgical margins, standardized adjuvant therapy, and close postoperative follow-up in reducing the risk of recurrence and metastasis. Further studies are warranted to optimize surgical approaches and comprehensive treatment strategies in order to improve long-term outcomes.

## Data Availability

The datasets presented in this study can be found in online repositories. The names of the repository/repositories and accession number(s) can be found in the article/supplementary material.
